# Molecular Dynamics Simulation of HIV Fusion Inhibitor T-1249: Insights on Peptide-Lipid Interaction

**DOI:** 10.1155/2012/151854

**Published:** 2012-05-22

**Authors:** A. M. T. Martins do Canto, A. J. Palace Carvalho, J. P. Prates Ramalho, Luís M. S. Loura

**Affiliations:** ^1^Departamento de Química, Escola de Ciências e Tecnologia, Universidade de Évora, Rua Romão Ramalho, 59, 7000-671 Évora, Portugal; ^2^Centro de Química de Évora, Universidade de Évora, Rua Romão Ramalho, 59, 7000-671 Évora, Portugal; ^3^Faculdade de Farmácia, Universidade de Coimbra, Pólo das Ciências da Saúde, Azinhaga de Santa Comba, 3000-548 Coimbra, Portugal; ^4^Centro de Química de Coimbra, Universidade de Coimbra, Rua Larga, 3004-535 Coimbra, Portugal

## Abstract

T-1249 is a peptide that inhibits the fusion of HIV envelope with the target cell membrane. Recent results indicate that T-1249, as in the case of related inhibitor peptide T-20 (enfuvirtide), interacts with membranes, more extensively in the bilayer liquid disordered phase than in the liquid ordered state, which could be linked to its effectiveness. Extensive molecular dynamics simulations (100 ns) were carried out to investigate the interaction between T-1249 and bilayers of 1-palmitoyl-2-oleoyl-phosphatidylcholine (POPC) and POPC/cholesterol (1 : 1). It was observed that T-1249 interacts to different extents with both membrane systems and that peptide interaction with the bilayer surface has a local effect on membrane structure. Formation of hydrogen bonding between certain peptide residues and several acceptor and donor groups in the bilayer molecules was observed. T-1249 showed higher extent of interaction with bilayers when compared to T-20. This is most notable in POPC/Chol membranes, owing to more peptide residues acting as H bond donors and acceptors between the peptide and the bilayer lipids, including H-bonds formed with cholesterol. This behavior is at variance with that of T-20, which forms no H bonds with cholesterol. This higher ability to interact with membranes is probably correlated with its higher inhibitory efficiency.

## 1. Introduction

Human immunodeficiency virus type 1 (HIV-1) fusion is mediated by a set of interactions involving cellular receptors and viral glycoproteins [1, 2]. Generally, viral attachment is thought to occur via an interaction between gp120 and CD4, along with chemokine receptors (such as CCR5 or CXCR4) that act as viral coreceptors for HIV-1 [3, 4]. Then, the gp41/gp120 oligomer suffers a conformational change that allows the fusion peptide sequence, located on the N terminus of gp41, to insert into the membrane of the target cell [1, 2]. The gp41 ectodomain forms the fusion-active state, which is believed to bring the viral and cellular membranes into closer proximity to facilitate membrane fusion [1–4].

Several peptides based on the C-region of HIV's gp41 have been used in clinical trials as possible HIV fusion inhibitors (FI) (reviewed in [5]). Among these is T-20 (also known as enfuvirtide). T-20 is a HIV FI approved for clinical use [6]. It is a 36-amino-acid peptide, homologous to the C-terminal region of HR2 of HIV-1 gp41 [7–10].

The elucidation of the core structure of gp41 has helped to understand the inhibitory activity of FI such as T-20 [9]. The peptide sequence (sequence 643–678 of HIV-1_LAI_ [7]) corresponded partially to the CHR region of gp41 and it would bind to the opposite NHR region, preventing the formation of the hairpin structure and ultimately, the fusion. Despite the therapeutic potency of T-20, it has met the emergence of resistant strains.

Similar peptides have been synthesized in order to achieve fusion inhibition without this setback. T-1249, a 39-aminoacid FI, composed of sequences derived from HIV-1, HIV-2, and simian immunodeficiency virus (SIV), is one such peptide [11]. Initial clinical trials with T-1249 have shown promising results: it is a more effective FI than T-20 even with a single daily administration instead of the two used for T-20 and retains activity against most T-20-resistant strains [7, 9, 11, 12].

The detailed molecular picture of the inhibitory mechanism promoted by these fusion inhibitors is still incomplete and differences in the effectiveness of these peptides are still a matter of debate. Both T-20 and T-1249 showed an efficient partition to zwitterionic bilayers; however, only T-1249 is able to interact/adsorb effectively to cholesterol-rich membranes, which may be the main cause of its improved efficiency (see [13, 14] for a detailed discussion). Both fluorescence spectroscopy data [13, 14] and simulation studies [15, 16] have shown that these peptides have the capacity to adsorb to/interact with the bilayer surface and suggest this as, at least, part of its mechanism of action. It was previously observed that T-1249 adsorbs (with more affinity than T-20) to the surface of both bilayers, without insertion in the studied timescale, presenting a helical structure (which has been related in the literature to increased efficiency in HIV fusion inhibition) and diffusing in the plane of the bilayer faster than the bilayer lipids (but slower than T-20) and retaining some rotational freedom [15].

In this work, we study the interaction of T-1249 with 1-palmitoyl-2-oleyl-phosphatidylcholine (POPC) and POPC/cholesterol (Chol) membranes using molecular dynamics (MD) simulations in the 100 ns time scale. Structure and behavior of all intervening molecular species are addressed. Our results mostly agree with the model of Veiga et al. [13] for the role of lipid bilayers in the mode of action of the peptide and may explain the relative more effective action of the peptide against HIV fusion when compared with T-20 [17], since high affinity to the bilayers implies high local concentrations of the peptide and thus the bilayer surface is able to act efficiently as a reservoir for the antifusion peptide.

## 2. Simulation and Analysis Details

**Figure 1:**
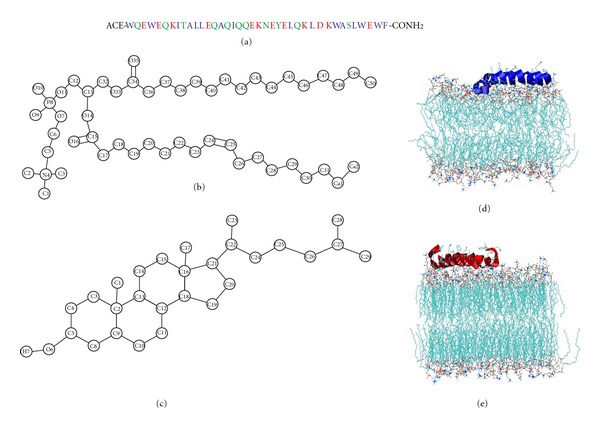
(a) T-1249 aminoacid sequence. (b) POPC structure and atom numbering. (c) Cholesterol structure and atom numbering. (d) T-1249/POPC final structure snapshot. (e) T-1249/POPC/Chol final structure snapshot. Reprinted with permission from [15]. Copyright 2010 Elsevier.

The initial *α*-helix model of T-1249 (see Figure 1(a) for primary structure) was built with the Arguslab 4.01 package [18] at neutral pH (as such, all acid aminoacid residues have a −1 charge, and all basic aminoacid residues have a +1 charge) and solvated in a cubic simulation box with SPC water [19], allowing for a distance between peptide and the box walls of 0.5 nm. POPC model molecules (Figure 1(b)) and their bonded and nonbonded parameters were downloaded from the Tieleman group web page (http://moose.bio.ucalgary.ca/index.php?page=Structures_and_Topologies). Cholesterol model molecules (Figure 1(c)) and their bonded and nonbonded parameters were taken from [20] and were downloaded from the GROMACS web page (http://www.gromacs.org/index.php?title=Download_%26_Installation/User_contributions/Molecule_topologies). Initial models of both membranes (POPC, 126 molecules; and POPC/Chol (1 : 1), 240 molecules in total; see Figure 1) were built. To this purpose, one POPC molecule (with mostly stretched and parallel acyl chains) from the downloaded POPC bilayer pdb file (together with one Chol molecule in the case of the T-1249/POPC/Chol system) was replicated to build custom size model bilayers using GROMACS model preparation packages [21, 22]. The latter was also used to perform all simulations. The GROMACS force field (which is a modified GROMOS87 force field) was used to describe all the interactions (see the GROMACS manual for details, ftp://ftp.gromacs.org/pub/manual/manual-3.3.pdf). Molecular dynamics of these systems, under the same conditions as the final MD runs (see below), were performed for at least 50 ns to ensure that the bilayers were equilibrated prior to the peptide inclusion in the system, losing memory of their initial structure in the process. Peptide and bilayer models were then combined, and the final structure obtained after 100 ns simulation of T-1249 in water was used as the initial structure of the simulations of the peptide interacting with the bilayer systems. The Z dimension of the simulation box was increased for this purpose, and the peptide molecule was positioned, with the helix's axis parallel to the bilayer surface (but with otherwise random orientation of its residues relative to the bilayer), at about 2.2–2.4 nm above the average position of the lipid P atoms of the top leaflet. The number of added SPC water molecules was sufficient to ensure full peptide and bilayer hydration in all systems (9602 water molecules added to the T-1249/POPC system, with average dimensions of 6.4 × 6.1 × 11.4 nm^3^, and 7398 water molecules added to the T-1249/POPC/Chol system, with average dimensions of 6.7 × 6.9 × 9.4 nm^3^). Systems with no added peptide were also simulated, and the main structural lipid properties were successfully verified for validation purposes, as described below. Prior to the production MD simulation, all systems underwent a steepest-descent energy minimization of the structure followed by a small MD run to properly allow the solvent molecules to adjust/relax around the peptide or membrane. Extensive MD simulations were then performed under constant number of particles, pressure (1 bar), temperature (300 K), and periodic boundary conditions. Pressure and temperature controls were carried out using the weak-coupling Berendsen schemes [23], with coupling times of 1.0 ps and 0.1 ps, respectively. Isotropic pressure coupling was used for the T-1249 simulation in water and semi-isotropic pressure coupling was used in all other simulations. All bonds were constrained to their equilibrium values using the SETTLE algorithm [24] for water and the LINCS algorithm [25] for all other bonds. Although our description of POPC is based on a united-atom model, both the peptide and cholesterol contain explicit H atoms. Very fast vibrations involving H atoms require the use of very small integration time steps, and therefore affect the efficiency of MD simulations. Constraining bond lengths allows the use of longer time steps, therefore improving efficiency [26].

The systems were simulated for 100 ns, with a time step of 2 fs. The long-range electrostatic interactions were calculated by the particle-mesh Ewald (PME) summation method [27]. A cut-off of 1.0 nm was used for both van der Waals and the PME direct-space component of electrostatic interactions. Analyses were carried out, mostly, using the GROMACS 3.3.3 analysis package [21, 22] with the exception of some membrane thickness calculations that were performed with the GridMat-MD program [28]. Errors were calculated according to the block method of Flyvbjerg and Petersen [29].

## 3. Results

### 3.1. Equilibration of the Membrane System

**Figure 2:**
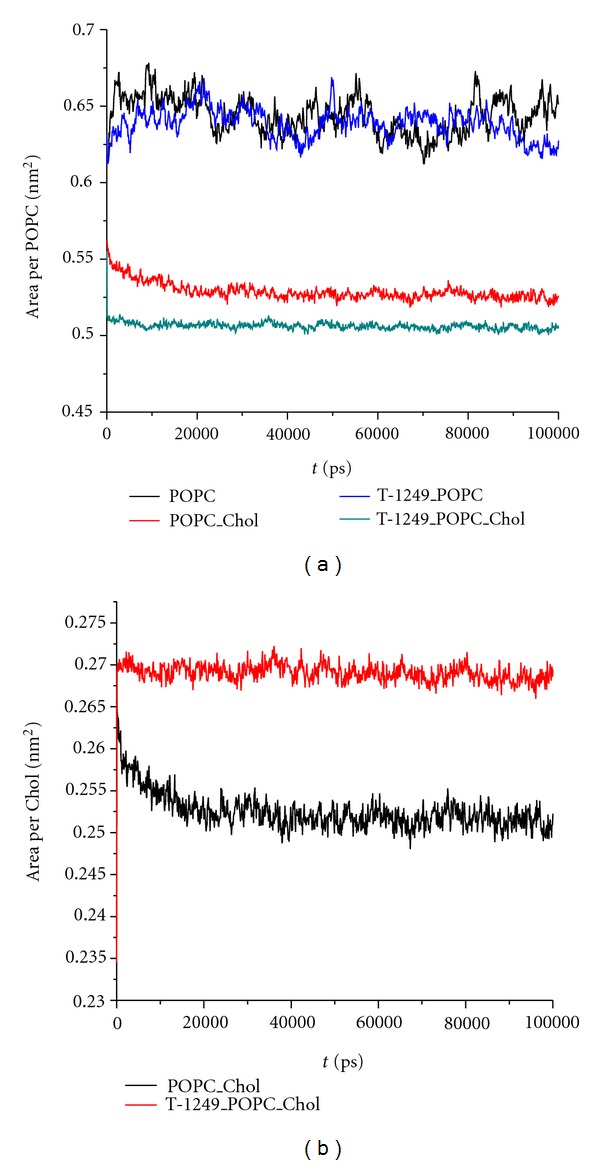
(a) Area per POPC time course. (b) Area per chol time course.

To evaluate the process of the systems' equilibration, time profiles of the surface area/POPC (Figure 2(a)) and surface area/Chol (Figure 2(b)) were calculated as in [30] (1) and recorded for the production simulation (100 ns; see section *cross-sectional area per lipid and membrane thickness *below for a detailed analysis of area/molecule values):

(1) $$\begin{array}{cc} A_{\text{POPC}} & =\frac{2A_{\text{box}}}{V_{\text{box}}-N_{W}V_{W}}\\ & \;\times \left[\frac{V_{\text{box}}-N_{W}V_{W}-xN_{\text{lipid}}V_{\text{Chol}}-V_{\text{T-}1249}}{\left(1-x\right)N_{\text{lipid}}}\right],\\ A_{\text{Chol}} & =\frac{2A_{\text{box}}V_{\text{Chol}}}{V_{\text{box}}-N_{W}V_{W}-V_{\text{T-}1249}}. \end{array}$$

In these equations, *A*_POPC_ is the cross-sectional area per POPC molecule, *A*_Chol_ is the cross-sectional area per Chol molecule, *A*_box_ is the area of *xy* plane of the simulation box, *V*_box_ is the simulation box total volume, *N*_*w*_ is the number of water molecules, *V*_*w*_ is the volume of the water molecule (*≈*0.030 nm^3^ at normal temperature and pressure conditions), *x* = 0.00 or 0.50 is the Chol mole fraction, *N*_lipid_ is the number of lipid molecules, *V*_chol_ is the volume of the Chol molecule (0.593 nm^3^) [30], and *V*_T-1249_ = 12.245 nm^3^ is the volume of the T-1249 molecule, determined from the T-1249 simulation in water by averaging *V*_T-1249_ = *V*_box_ − *N*_*w*_ × *V*_*w*_ for the last 25 ns of the simulation.

The surface area per lipid is a slowly converging parameter of MD simulation, but its average value was stable over the final 80 ns of the simulation, which led us to the conclusion that the simulated systems had reached a steady state after 20 ns of simulation (Figure 2).

### 3.2. Peptide General Behavior

In the starting configuration, T-1249 was placed at approximately 2.2–2.4 nm above the membrane surface, defined as the average of the *z* positions of all POPC P atoms. In all cases T-1249 assumes a helical structure preferring the *π*-helix in all cases as described earlier [15] and also observed for T-20 [16], suggesting that this trend may be paramount for function. It was previously observed, in both membrane systems, that the the peptide approaches the membrane surface and adsorbs to it in <20 ns [15]. It was also previously observed that the peptide's adsorbed position, in the equilibrium, is approximately parallel to the membrane surface. In both simulated systems it assumes a tilt (defined as the time average of the angle between the vector defined by the 2nd and 38th C*α* and the plane parallel to the membrane surface) of 2.1° ± 1.5° for the T-1249/POPC/Chol bilayer system and of 8.6° ± 3.1° for the T-1249/POPC system [15]. The POPC bilayer is in the liquid disordered state, with increased free volume relative to the POPC/Chol liquid ordered membrane. This is probably why its C-terminus penetrates below the membrane surface, as described earlier. This does not occur in the T-1249/POPC/Chol system, where the peptide does not penetrate the bilayer and assumes an orientation more parallel to the membrane surface (as also evident from the tilt angles and in the typical snapshots of Figures 1(d) and 1(e)).

**Figure 3:**
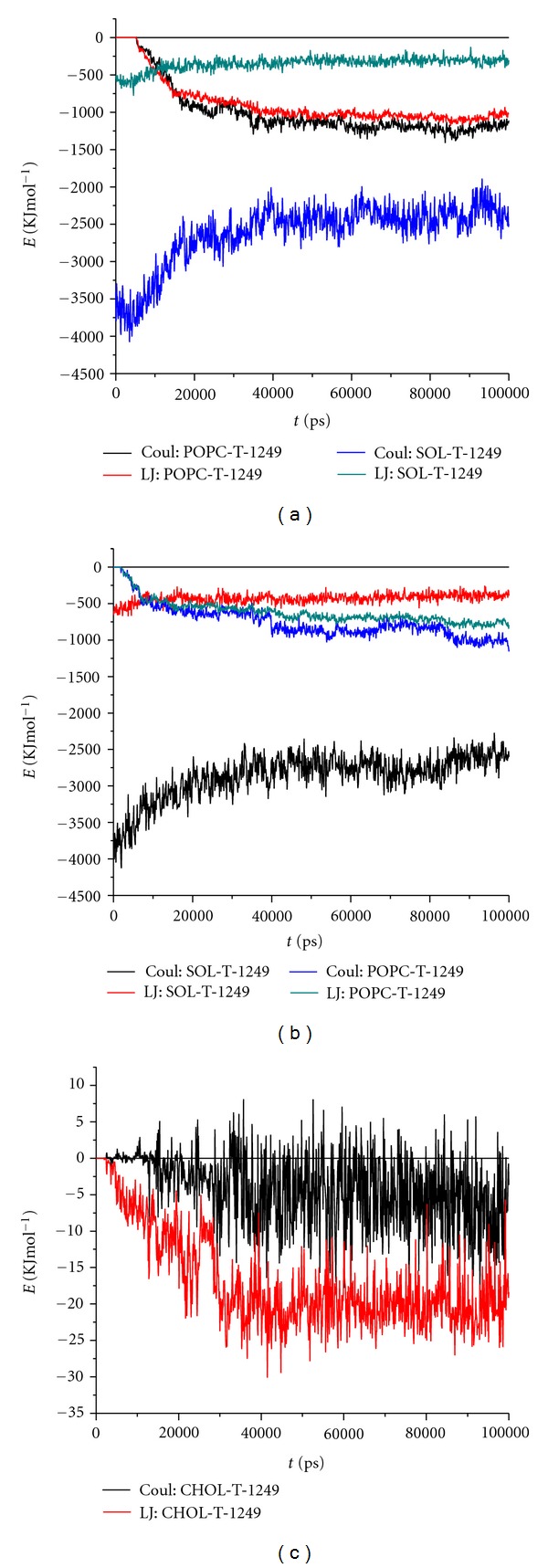
Time variations of Coulomb and Lennard-Jones peptide/POPC and peptide/solvent interaction energies, in the POPC (a) and POPC/Chol (b) systems. (c) Time variations of Coulomb and Lennard-Jones peptide/Chol interaction energies in the T-1249/POPC/Chol system.

To get further insight on the driving force behind binding of the peptide to the bilayers, time variations of both Coulomb and Lennard-Jones peptide/lipid and peptide/solvent interaction energies are shown in Figure 3. Inspection of these plots reveals a lag-time of 2–5 ns in which peptide/lipid interaction energy is essentially zero. During this period, the peptide is too distant from the bilayers to be able to interact with them and diffuses in the water medium. This diffusion eventually leads the peptide to the regions in the box where the presence of lipid can be felt. From this point on, both T-1249/POPC Coulomb and Lennard-Jones interaction energies decrease gradually and conversely for the solvation energies. In the early stages of interaction, the two terms have similar magnitude in both systems. However, from 18–20 ns onwards, the Coulomb term becomes the largest in absolute terms in both systems, probably reflecting peptide helix/lipid headgroup reorientation, with concomitant formation of favorable ionic and H-bond interactions. For comparison purposes, T-20 interacting with model membranes [16] were revisited and average interaction energies were calculated for the 100 ns of each simulation. The same was calculated for the T-1249 simulations. Interacting with both POPC bilayer and the POPC/Chol bilayer, T-1249 has lower (more negative) interaction energies with all the membrane components (view Table 1 where a summary of T-20 results is compared with T-1249 results), which suggests a stronger interaction with the bilayers than observed with T-20 [16]. Regarding the T-1249/POPC/Chol system, and in contrast with previously published results on T-20 behavior [16], it is noteworthy that (i) T-1249/Chol interaction is not negligible and (ii) the Lennard-Jones energy component of the T-1249-Chol interaction is higher than the Coulomb component.

### 3.3. Peptide Interaction with Bilayers: Radial Distribution Functions (RDF)

**Figure 4:**
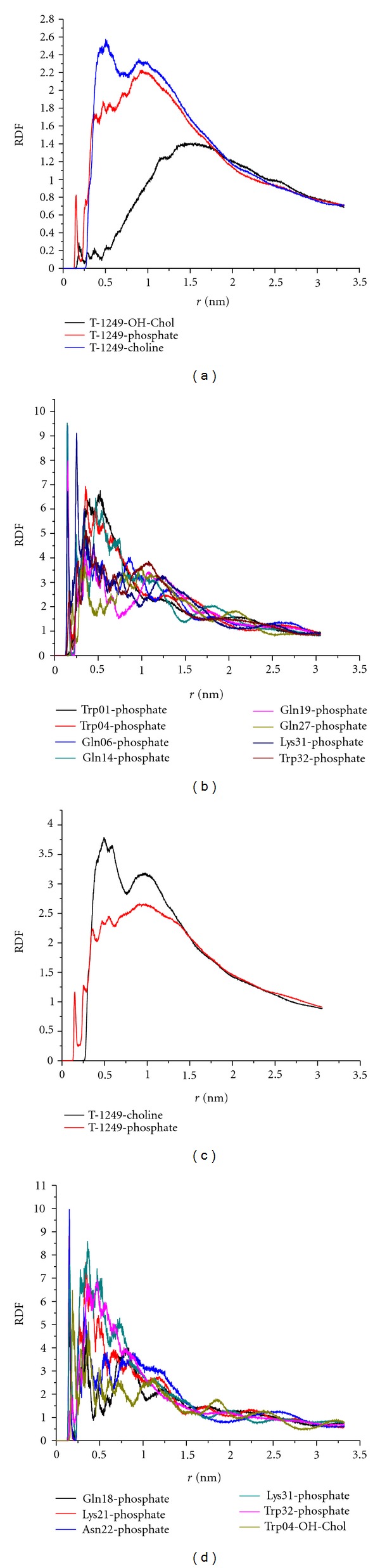
Radial distribution functions. (a) RDFs between T-1249 and the phosphate, choline and OH-Chol groups in the T-1249/POPC/Chol system. (b) RDFs between selected aminoacid residues and POPC's phosphate groups in the T-1249/POPC system. (c) RDFs between T-1249 and the phosphate and choline groups in the T-1249/POPC system. (d) RDFs between selected aminoacid residues and POPC's phosphate and OH-Chol groups in the T-1249/POPC/Chol system.

Radial distribution functions (RDFs) were calculated between all the T-1249 atoms and all the atoms of the phosphate (O7, P8, O9, O10, and O11) and choline (C1, C2, C3, and N4) groups, and, exclusively in the POPC/Chol bilayer, also the Chol hydroxyl group (O6 and H7) (Figures 4(a) and 4(c)), for the last 25 ns of the simulation time.

The RDFs for the choline group have the highest densities within each system (higher in the T-1249/POPC system than in the T-1249/POPC/Chol system in all cases) and the distribution function with the lowest density is the one of T-1249 with the Chol's hydroxyl group, albeit higher than in the T-20 case [16], and with a narrow peak at *≈*0.15 nm inexistent in the T-20 case [16]. Although T-1249/phosphate RDFs have lower densities than those of the choline (but still higher in the POPC system than in the POPC/Chol bilayer), T-1249 appears consistently at a shorter distance from the phosphate group than all the others (*≈*0.15 nm). This distance and the narrowness of its peak suggest a very specific interaction between certain aminoacid residues of the peptide and the phosphate group.

For further insight, RDFs between individual aminoacid residues and the mentioned lipid polar groups were calculated. Trp04, Gln18, Lys21, Asn22, Lys31, and Trp32 were found to be the main contributors to the 0.15 nm density peak in the POPC/Chol system (Figure 4(d)), whereas in the POPC system the main contributors are Trp01, Trp04, Gln06, Gln14, Gln19, Gln27, Lys31, and Trp32 (Figure 4(b)). These aminoacid residues are all capable of acting as an H donor or acceptor in an H bond and the distance of the interactions is supportive of such hypothesis.

The fact that the T-1249/choline RDF has a very broad peak (in both systems) at ~0.6 nm is probably related to the interfacial location of the choline moiety and its accessibility to the solvent and hence to the peptide itself, rather than to a specific T-1249/choline interaction. Interaction with the cholesterol hydroxyl group appears to be stronger than the corresponding interaction between T-20 and Chol [16]. In POPC/Cholesterol mixtures, Chol is expected to be protected from water by the PC headgroup (the so-called umbrella effect [31]), but even so, T-1249 (unlike T-20) is able, in the time scale studied, to position at least one aminoacid residue in a position that allows it to interact with Chol molecules in such a proximal way.

### 3.4. Peptide Interaction with Bilayers: H Bonds

Following the previous section, formation of H-bonds between individual residues of T-1249 (as well as on the whole) and relevant groups in the bilayer systems were investigated. For this analysis, an H bond for a given donor-H-acceptor triad was registered each time the donor–acceptor distance was <0.35 nm and the H-donor-acceptor angle was <30°.

**Figure 5:**
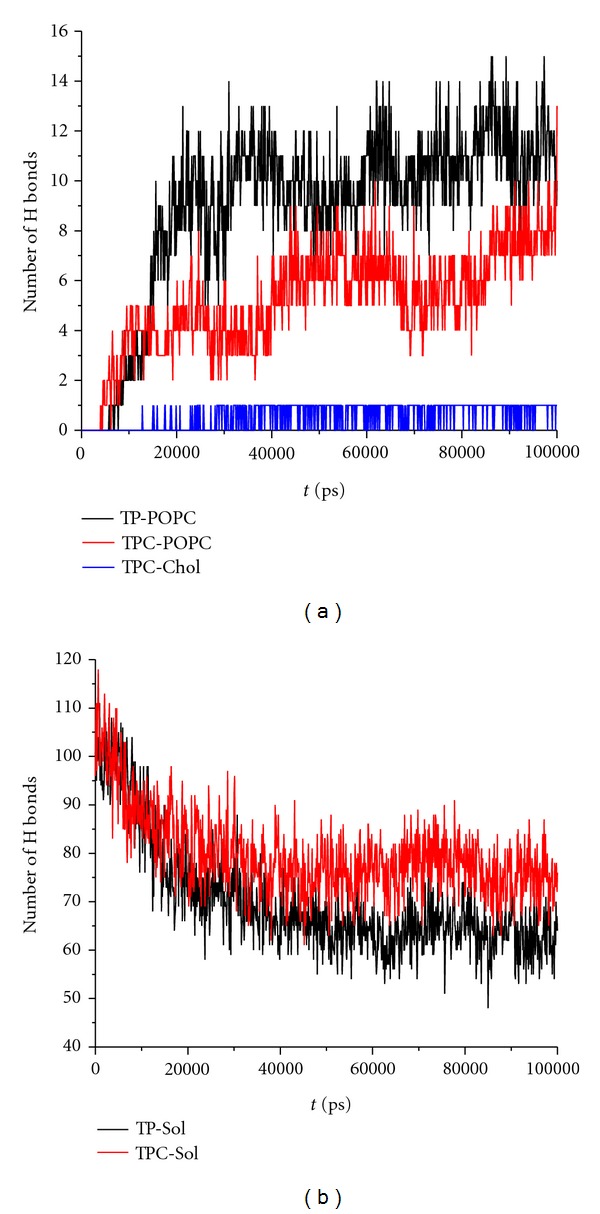
(a) Time course of the number of H bonds formed between T-1249 and POPC molecules in T-1249/POPC system (black), T-1249 and POPC molecules in the T-1249/POPC/Chol system (red), and T-1249 and chol molecules in the T-1249/POPC/Chol system (blue). (b) Time course of the number of H bonds formed between T-1249 and solvent molecules in T-1249/POPC system (black) and T-1249/POPC/Chol system (red). (where TP stands for T-1249/POPC System and TPC stands for T-1249/POPC/Chol system).

Figure 5 shows the time variation of the number of H-bonds formed between T-1249 and the POPC molecules and T-1249 and the water molecules. T-1249 is capable of binding to both bilayers via H bonds and their number generally increases with time during the 100 ns of the simulation. Adsorption gives rise to a steep increase in the number of H bonds formed between the peptide and the bilayer in the POPC system for 10 ns < *t* < 20 ns. The increase in number of T-1249/POPC H bonds is much more gradual in the POPC/Chol bilayer. The number of H bonds in the T-1249/POPC/Chol system is clearly lower than in the T-1249/POPC system during most of the simulated time, and this significant difference cannot solely be explained by the small difference in POPC molecules per leaflet (63 in the POPC bilayer and 60 in the POPC + Chol bilayer). Also, upon adsorption, the number of H bonds formed with water molecules decreases significantly. This decrease even surpasses the number of H bonds formed with the bilayer. This appears to be caused by the adsorption process itself, since it occurs simultaneously and stabilizes with it. Hence the total number of H bonds T-1249 is able to form decreases by *≈*27% in the POPC system and 22% in the T-1249/POPC/Chol system. The lower ability of T-1249 to interact via H bonds with the POPC/Chol bilayer is thus also reflected by a lower influence in the decrease in H bond formation with the water molecules.

Individual residues were analyzed (last 25 ns of the simulation) to determine which ones were responsible for the formation of H bonds with the bilayer (Table 2). T-1249 interacts via H bonds mainly with the phosphate O atoms and the carbonyl O16 atom and Chol's OH in the POPC/Chol bilayer. Interaction with the other POPC ester and carbonyl O atoms is negligible. In the POPC bilayer, the H bond donor residues that contribute to H bond formation are Trp01, Gln02, Trp04, Gln06, Gln14, Gln18, Gln19, Gln27, Lys31, Trp32, Trp36 and Trp38, which concurs with the RDFs results. In the POPC/Chol bilayer, Trp04, Gln16, Gln18, Lys21, Asn22, Gln27, Lys31, Ser34, Trp36, and Trp38 contribute to H bonding to POPC, which also agrees with the RDFs results. Contrary to T-20′s behavior, T-1249 is able to form H bonds with Chol [16]. In both systems, some of the donors appear in clusters: Trp01-Gln02, Gln18-Gln19, Lys31-Asn32 in the T-1249/POPC system, and Lys21-Asn22 in the T-1249/POPC/Chol system. This probably stems from the fact that formation of an H bond between a given residue and phosphate O atoms contributes in turn to approximate and/or provide adequate orientation of the neighboring residues relative to the phosphate group, facilitating their own involvement in H bond formation. The peptide H-bond donors and acceptors span most of the peptide helix length in both systems, thus allowing for an early and almost parallel adsorption to the bilayers.

### 3.5. Cross-Sectional Area per Lipid and Membrane Thickness

The cross-sectional area per lipid (POPC or Chol) was calculated as reported in [30] with minor modifications to take into account the volume occupied by the peptide when present. Briefly, both parameters were calculated according to (1). The POPC/Chol bilayers, when compared with the POPC bilayers, showed a lower area per POPC as expected due to the Chol's condensing effect [30, 32, 33].

Membrane thickness was determined as the average of the distance between P atoms of different monolayers (P-P distance). Membrane thickness values are difficult to compare to experimental results because a definitive definition of bilayer thickness is still lacking [33]. Overall the POPC/Chol bilayers are thicker than the POPC bilayers, as expected due to the acyl-chain ordering induced by Chol [30, 33]. The presence and interaction of the T-1249 peptide with the model membranes have the same effect in both cases albeit to different extents: it induces a decrease in the membrane thickness of about 0.5% to the POPC membrane and of about 3.8% to the POPC/Chol membrane (Table 3). Upon adsorption with the POPC or POPC/Chol bilayers, T-1249 induces a decrease in the area per POPC of about 1.8% and 3.9%, respectively (Table 3).

Contrary to POPC surface area, the area/Chol molecule increases approximately 6.7% upon T-1249 adsorption to the model POPC-Chol membrane (Table 3). This implies that globally the area of the POPC/Chol bilayer is only slightly changed (*≈*0.5% decrease) upon adsorption of the peptide. The fact that, calculating the average area/POPC and area/Chol using (1) leads to a decrease in the former, and an increase in the latter, should be viewed with caution, because as there is a strong *Z*-dependence between the cross-sectional areas of PC and cholesterol, the average area per phospholipid and area per cholesterol in binary mixtures are in fact poorly defined parameters [32].

**Figure 6:**
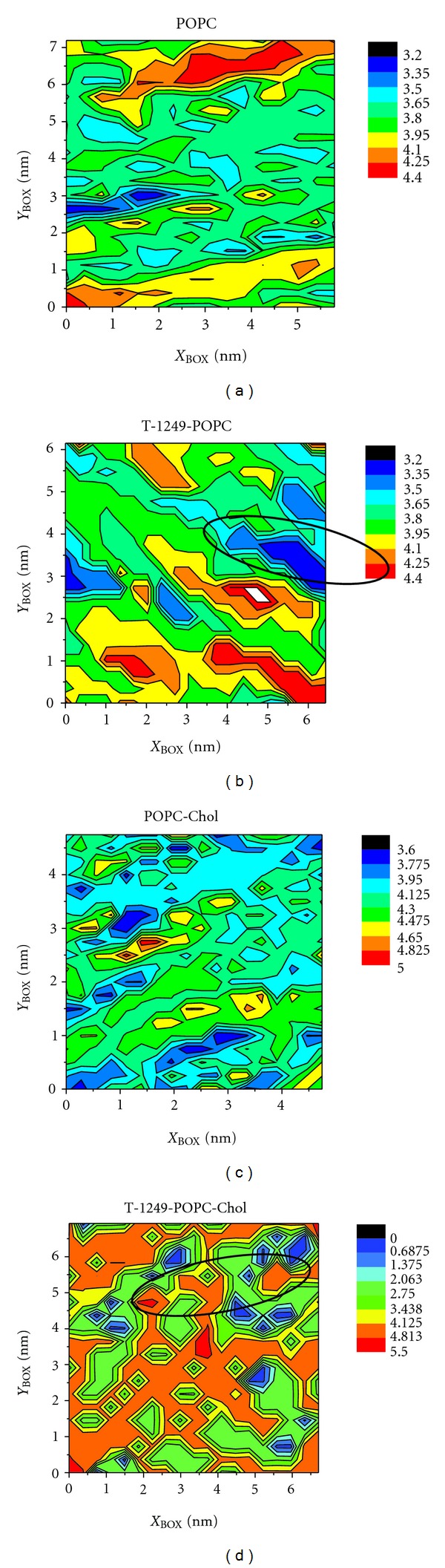
Membrane thickness contour plot of the last configuration in each system; (a) POPC system, (b) T-1249/POPC system, (c) POPC/Chol system and (d) T-1249/POPC/Chol system. Peptide position is depicted as a black ellipse.

The POPC bilayer is a fluid membrane and upon adsorption, T-1249 semi penetrates the membrane surface forming a crater-like burrow around itself as clearly shown in the 2D plot of local bilayer thickness (calculated using the Gridmat-MD program [28]) shown in Figure 6. This plot represents a 2D map of the bilayer in which the local bilayer thickness is represented across the bilayer plane. Some POPC molecules, the ones directly below the peptide, are pushed towards the bilayer core (Figure 6). As a result, this compression creates a concavity in the top leaflet that ultimately leads to an average decrease in bilayer thickness, considering the entire bilayer (Table 3). This effect was also observed in the T-1249/POPC/Chol system but to a lesser extent (Figure 6), hence the lesser decreases in membrane thickness upon peptide adsorption.

**Figure 7:**
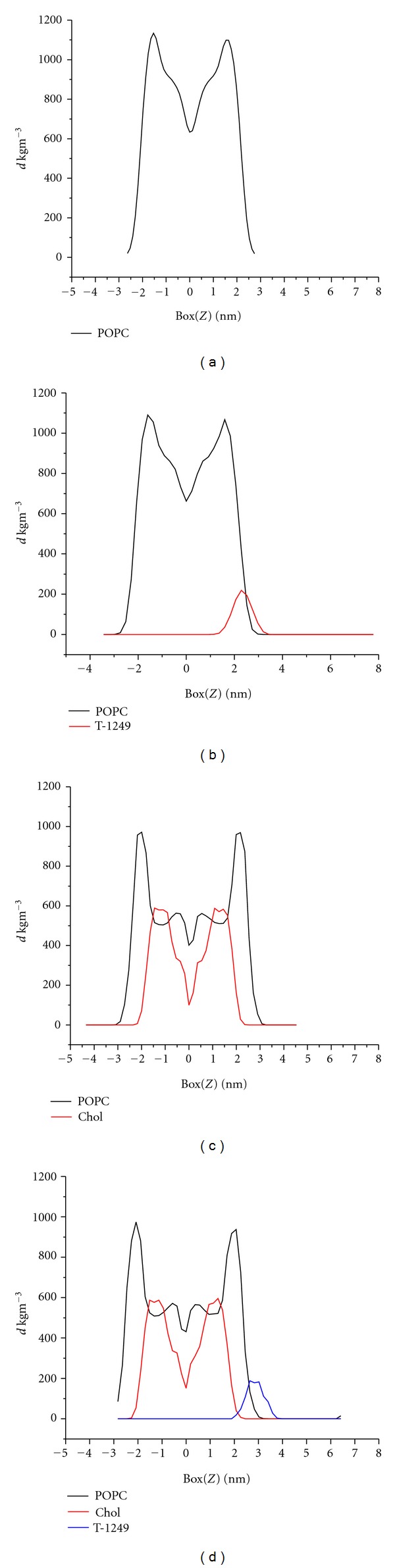
Mass density profiles. (a) POPC system, (b) T-1249/POPC system, (c) POPC/Chol system, and (d) T-1249/POPC/Chol system.

Profiles of the mass density were calculated for the molecules present in the bilayer systems in study (averaged over the last 25 ns of the simulation) along the normal to the membrane plane as shown in Figure 7. The compression caused by peptide interaction with the top leaflet, of both systems, is also visible in the POPC density in this region (but not in the cholesterol density). The top leaflet, upon which the peptide adsorbs, consistently has a lower POPC peak density and a slight POPC profile distortion when compared with the bilayers without peptide.

### 3.6. Order Parameters

The order parameter tensor, *S*, is defined as:

(2) $$S_{ab}=\frac{1}{2}\langle 3\cos\left(\theta_{a}\right)\cos\left(\theta_{b}\right)-\delta_{ab}\rangle \;a,b=x,y,z,$$

where *θ*_*a*_ (or *θ*_*b*_) is the angle made by *a* ^th^ (or *b* ^th^) molecular axis with the bilayer normal and *δ*_*ab*_ is the Kronecker delta (〈〉 denotes both ensemble and time averaging). In our simulations using a united atom force field, the order parameter for saturated and unsaturated carbons *S*_CD_ can be determined using the following relations [34]:

(3) $$\begin{array}{c} -S_{\text{CD}}^{\text{Sat}}=\frac{2}{3}S_{xx}+\frac{1}{3}S_{yy},\\ -S_{\text{CD}}^{\text{Unsat}}=\frac{1}{4}S_{zz}+\frac{3}{4}S_{yy}+\frac{\sqrt{3\;}}{2}S_{xy}. \end{array}$$

−*S*_CD_ may vary between 0.5 (full order along the bilayer normal) and −0.25 (full order along the bilayer plane), whereas −*S*_CD_ = 0 denotes isotropic orientation. Due to the slow convergence of this parameter [35], analysis was restricted to the last 10 ns of the simulations.

**Figure 8:**
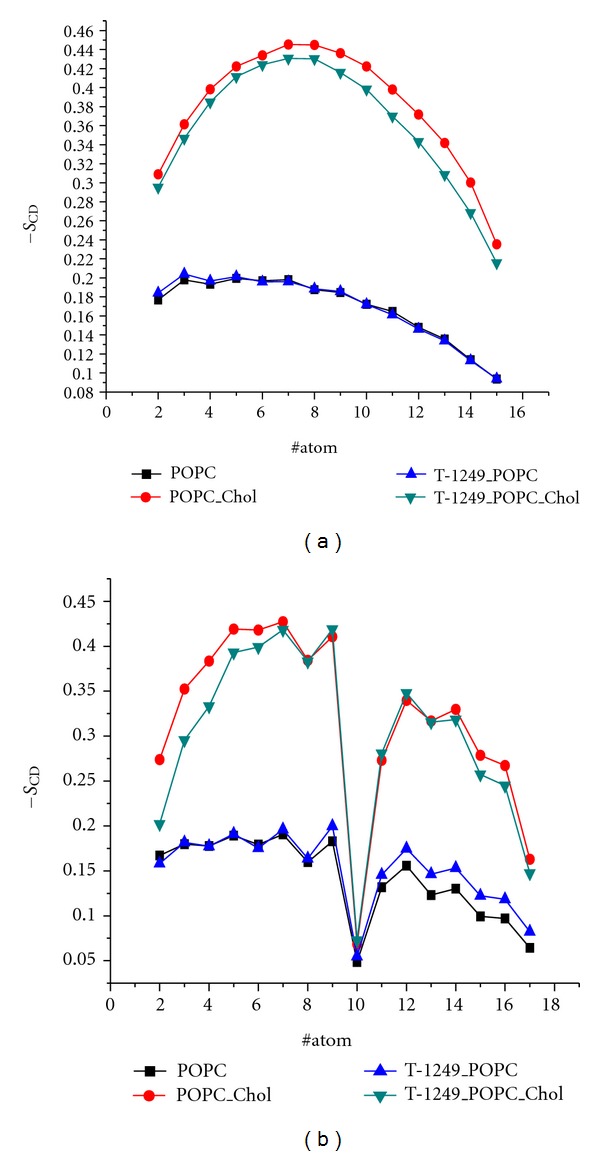
−*S*_CD_ order parameters of *sn*-1 (a) and *sn*-2 (b) acyl chains.

−*S*_CD_ profiles along the *sn*-1 and *sn*-2 chains in POPC, POPC/Chol, T-1249/POPC, and T-1249/POPC/Chol systems are shown in Figure 8. In the POPC/Chol bilayer, T-1249 adsorption generally evokes a decrease in −*S*_CD_ values in both acyl chain C atoms. In the POPC bilayer the C2-C4 atoms of the *sn*-1 acyl chain suffer a slight increase in − *S*_CD_, which can be a result of a more local and intense interaction between the bilayer and the peptide (which, in this system, is located deeper within the bilayer interface and closer to the bilayer hydrophobic core than in the T-1249/POPC/Chol system), as this effect is not observed in the POPC/Chol bilayer in which peptide adsorption induces chain disordering. Also atoms C9 and C11-C17 suffer an increase in − *S*_CD_. In the POPC/Chol bilayer the general effect of peptide adsorption, as stated earlier, is a decrease in − *S*_CD_ values in both acyl chain C atoms. This effect is more pronounced in the *sn-*1 chain and the *sn*-2 acyl chain's C2-C7 carbons.

## 4. Discussion

100 ns molecular dynamics simulations of solvated bilayers (POPC, in the liquid disordered phase, and POPC/Chol 1 : 1 liquid ordered phase) were performed for comparison purposes, as stated earlier. Those bilayers were also analyzed and several parameters where determined both for validation purposes and comparison with the peptide simulations. Our results for the cross-sectional area per POPC (Table 3) agree with the experimental values of 0.65 nm^2^ (*T *= 298 K; Lantzsch et al., [36]), 0.64 nm^2^ (*T *= 298 K; Konig et al., [37]), and 0.63 nm^2^ (*T *= 297 K; Smaby et al., [38]), as well as with those obtained from MD simulations by Bockmann et al. [35] (*T* = 300 K, *a* = 0.655 nm^2^), Mukhopadhyay et al. [39] (*T* = 298 K, *a* = 0.62 nm^2^), Gurtovenko and Anwar [40] (*T* = 310 K, *a* = 0.65 nm^2^), and Pandit et al. [41] (*T* = 303 K, *a* = 0.63 nm^2^). Order parameters were also calculated for both acyl chains of POPC (Figure 8). The profiles obtained agreed with both experimental (e.g., [42, 43]) and simulated (e.g., [35, 40, 44]) data. Calculated lateral lipid diffusion coefficients (not shown) agreed with values obtained both from NMR experiments [45, 46] and MD simulations [35]. Together, these findings validate our bilayer model systems for the study of interaction with T-1249. It has been proposed recently that a better description of the cis-double bond in unsaturated acyl chains may be achieved by a parameterization that accommodates skew states [47, 48], which is absent in our model. Although its inclusion could potentially lead to a slightly more accurate description of the lipid bilayer systems (e.g., slightly lower order parameters for the POPC/Chol bilayer), because our main focus lies on the relative peptide effect on the bilayers and not the absolute properties of the bilayers themselves, the absence of these forcefield improvements does not hamper our discussion and conclusions.

This and previous studies [15] show that HIV fusion inhibitor peptide T-1249 interacts with POPC (liquid disordered phase) and POPC/Chol 1 : 1 (liquid ordered phase) bilayers to a higher extent than T-20 [15, 16]. This was verified experimentally by Veiga et al. [13, 14]. These authors measured the variation of peptide fluorescence intensity (from the five Trp residues) and determined a lipid/water partition coefficient of *K*_*p*_ = (5.1 ± 0.7) × 10^3^ in POPC, using a formalism of distribution between aqueous and lipid phases (valid assuming peptide insertion in the bilayer; see below for discussion of the latter hypothesis), significantly higher than that of T-20 in the same system ((1.6 ± 0.1) × 10^3^). Veiga et al. verified significant adsorption of T-1249 to chol rich membranes/domains with *K*_*a*_ = 3.1 × 10^3^ and *K*_*a*_ = 4.6 × 10^3^ for 18% and 25% chol, respectively, in POPC/Chol membranes, indicating a strong enough interaction with lipid membranes for those authors to hypothesize an important role of membranes in T-1249 mode of action. These authors discuss the importance of binding to ordered POPC-Chol bilayers as possibly correlating to the increased efficiency of T-1249 relative to other inhibitors such as T-20. The present work supports those observations since no membrane penetration was observed, in the studied time scale, but adsorption of the peptide was observed in approximately the same time scale in which it occurs in POPC liquid crystalline bilayers. This behavior differs significantly from that of T-20 interacting with POPC/Chol membranes, in which interaction was much weaker and delayed [16]. However, no effective peptide insertion is observed in the timescale of our simulations, and the peptide stays adsorbed at the interface, in this behaving in a similar way to T-20 [16].

The absence of peptide insertion in our observations does not necessarily mean that the partition treatment of Veiga et al. and their model of the involvement of lipid membranes in the peptide fusion inhibitor's mode of action, both of which assume peptide insertion in Chol-free bilayers, are incorrect, because peptide insertion cannot be ruled out for larger timescales, inaccessible to MD simulations. It could be argued that peptide insertion from a predominantly helical initial structure such as in our model could be more difficult than insertion from a mainly random conformation such as reported for T-1249 by Veiga et al. from circular dichroism (CD) measurements [13]. However, it should be noted that recovery of secondary structure from CD data is an inverse problem (ill-poised by nature), and most algorithms used for this effect only differentiate three general types of secondary structure. Our determination of a majority helical conformation both in solution and in interaction with bilayers [15] is not subject to this restriction. In our study, we considered the protonation states of each aminoacid residue as expected for neutral pH, yielding a global peptide charge of −4 [13]. One cannot rule out that, for other pH values and protonation states, eventual differences in secondary structure would allow for more rapid bilayer insertion.

As observed for T-20, our simulations of T-1249 interacting with model membranes show that peptide adsorption is clearly related to the formation of H bonds between some peptide residues and mainly, but not exclusively, the POPC phosphate O atoms. The fact that a stronger interaction is observed for T-1249 in both systems correlates with the larger average number of such H bonds in this system, with T-1249 (T-1249 is able to establish an average of more 15.3% and 26.5% H bonds with the POPC and POPC/Chol bilayers, respectively, than T-20 forms [16]). This increase, namely in the interaction between T-1249 and the POPC/Chol bilayer, does not appear to be due to a single aminoacid but to a wide set of aminoacid residues, (wider than in the T-20 case [15]) spread more evenly throughout the length of the peptide, and thus promoting a more stable adsorption/interaction of the peptide with the bilayer. The higher number of H bonds that T-1249 forms with both bilayers also correlates with the slower dynamics it assumes, as reported earlier [15, 16].

## 5. Concluding Remarks

In summary, despite the obvious limitations concerning the sampling timescale (which precluded the study of slower interaction processes, such as eventual peptide insertion) and simulation of a single peptide molecule (with obvious consequences in terms of statistics; a possible way to circumvent this would be averaging over a number of shorter simulations with different initial structures), our simulations provide detailed insight on the nature of the interaction of T-1249 with model membranes, indicating that the peptide adsorbs (with more affinity than T-20) to the surface of both POPC and POPC/Chol 1 : 1 bilayers (less strongly in the latter case but still much more strongly than observed for T-20 in the same system), without insertion in the studied timescale. T-1249 is able to establish H bonds with both POPC and Chol, and although the number of H bonds is higher in the pure POPC system, as in the case with T-20 [16], T-1249 is able to establish more H bonds with the POPC/Chol bilayer than T-20, including H bonds with Chol, which were not observed in the T-20 case. This could explain the peptide's higher affinity to this bilayer system. These observations mostly agree with the model of Veiga et al. [13] for the role of lipid bilayers in the mode of action of the peptide and may explain the relative higher efficiency of the peptide against HIV fusion when compared with other similar, first generation peptides [13–16] since high affinity to the bilayers implies high local concentrations of the peptide, and the bilayer surface is thus able to act efficiently as a reservoir for the antifusion peptide.

While the present work is useful in its characterization of the main determinants of T-1249-bilayer interaction, in order to obtain a more thorough description of the energy barriers involved, calculations of the free energy profile of the peptide across the membrane systems are needed. Due to the high computation effort involved, these calculations could not be carried out at this stage. However, additional simulation work to that effect is currently being devised at our laboratory.

## Figures and Tables

**Table 1:** Lennard-Jones and Coulomb interaction energies between the peptides, T-20 and T-1249, and the other system components (POPC, Chol and Water, depicted as SOL). The results are averaged over all the 100 ns of the simulations so as to encompass all the aspects of the peptide's behavior. Percentages of variation, comparing T-20 with T-1249 behavior, are also presented with the T-1249 results.

	LJ/KJmol^−1^		Coulomb/KJmol^−1^	
	POPC	POPC/Chol	POPC	POPC/Chol
T-20-POPC	−722.48 ± 57.00	−353.41 ± 29.60	−959.43 ± 77.36	−385.64 ± 42.03
T-20-Chol		−14.80 ± 1.82		−2.12 ± 0.26
T-20-SOL	−389.08 ± 11.91	−447.01 ± 8.29	−1970.25 ± 97.43	−2907.67 ± 36.50
T-1249-POPC	−885.70 ± 65.80 **(−22.6%)**	−614.37 ± 36.74 **(−73.8%)**	−989.45 ± 79.39 **(−3.1%)**	−746.55 ± 50.57 **(−93.6%)**
T-1249-Chol		−16.86 ± 1.33 ** (−13.9%)**		−4.13 ± 0.52 **(−94.8%)**
T-1249-SOL	−351.62 ± 16.76 **(+9.6%)**	−429.61 ± 8.68 **(3.9%)**	−2611.35 ± 88.12 **(−32.5%)**	−2861.01 ± 61.88 **(1.6%)**

**Table 2:** Average number of H bonds between T-1249 aminoacid residues (or some relevant aminoacid residues) and relevant atoms in the bilayer structure.

	Phosphate O atoms		O16 (carbonyl O atom of *sn-2* chain)		OH Chol
	POPC	POPC : Chol	POPC	POPC : Chol	POPC : Chol
T-1249	9.62 ± 0.63	5.60 ± 1.09	1.63 ± 0.22	0.75 ± 0.13	0.86 ± 0.10
Trp 01	0.44 ± 0.33	—	—	—	
Gln 02	0.15 ± 0.29	—	—	—	
Trp 04	1.02 ± 0.05	0.05 ± 0.04	—	—	0.86 ± 0.10
Gln 06	1.01 ± 0.04	—	—	—	
Gln 14	1.01 ± 0.02	—	—	—	
Gln 16	—	0.02 ± 0.03	—	—	
Gln 18	1.01 ± 0.02	0.87 ± 0.31	—	—	
Gln 19	1.00 ± 0.00	—	—	—	
Lys 21	—	1.02 ± 0.04	—	0.75 ± 0.13	
Asn 22	—	0.98 ± 0.04	—	—	
Gln 27	0.65 ± 0.17	0.06 ± 0.13	—	—	
Lys 31	1.19 ± 0.15	1.46 ± 0.66	—	—	
Trp 32	0.99 ± 0.07	—	—	—	
Ser 34	—	0.01 ± 0.03	—	—	
Trp 36	—	0.94 ± 0.37	0.81 ± 0.13	—	
Trp 38	0.02 ± 0.05	0.03 ± 0.05	0.83 ± 0.15	—	

**Table 3:** Cross-sectional area per lipid in all systems under study and their respective membrane thickness.

	Lipid	Area per lipid/nm^2^	Membrane thickness/nm
POPC	POPC	0.645 ± 0.011	3.82 ± 0.08
POPC : CHOL	POPC	0.526 ± 0.003	4.59 ± 0.03
	CHOL	0.252 ± 0.001	
T-1249-POPC	POPC	0.634 ± 0.009	3.80 ± 0.05
T-1249 + POPC + CHOL	POPC	0.505 ± 0.002	4.43 ± 0.11
	CHOL	0.269 ± 0.001	
